# The Digital Era Heralds a Paradigm Shift in Dentistry: A Cross-Sectional Study

**DOI:** 10.7759/cureus.53300

**Published:** 2024-01-31

**Authors:** Mahesh Suganna, Ramesh P Nayakar, Aisha A Alshaya, Rahaf O Khalil, Shahad T Alkhunaizi, Kawssar T Kayello, Luluwah A Alnassar

**Affiliations:** 1 Department of Prosthodontics, College of Medicine and Dentistry, Riyadh Elm University, Riyadh, SAU; 2 Department of Prosthodontics and Crown and Bridge, Karnataka Lingayat Education Vishwanath Katti Institute of Dental Sciences, Karnataka Lingayat Education Academy of Higher Education and Research, Belagavi, IND; 3 Department of Dentistry, College of Medicine and Dentistry, Riyadh Elm University, Riyadh, SAU

**Keywords:** clinical decision-making, education, professional awareness, cad/cam technology, dentistry, digital technology

## Abstract

Background

The transformation of dental practice from conventional methods to digital technology has been widely discussed. This study aimed to examine the awareness, understanding, and attitudes towards the use of digital technology in dentistry, with a particular focus on computer-aided design/computer-aided manufacturing (CAD/CAM) technology.

Methods

A cross-sectional study was conducted, involving a questionnaire distributed to a diverse group of participants from the dental field. The questionnaire covered aspects of digital technology in dentistry, including awareness, perceived usefulness, understanding of CAD/CAM technology, perceived benefits and shortcomings, system awareness, and the impact on clinical decision-making.

Results

Almost all participants (99.3%) reported being aware of digital technology in dentistry. The perceived utility of digital technology varied widely, but it was considered particularly useful for specific dental procedures. Most of the respondents (948 out of 953) were aware of CAD/CAM technology, and many acknowledged its benefits, including fewer appointments, less chairside time, and greater precision. However, high cost, lack of knowledge, and preference for traditional methods were identified as barriers to adoption. Most participants believed that CAD/CAM would influence clinical decision-making and expressed interest in integrating it into their regular workflow. A majority had attended CAD/CAM training programs and believed there was a need to increase education on CAD/CAM during undergraduate and postgraduate courses. While a significant majority agreed that digital technology was the future of dentistry, a substantial number also expressed reservations.

Conclusion

The study concluded that there is a high level of awareness and readiness to adopt digital technology in dentistry. However, its perceived utility varied among participants, and several barriers to adoption were identified, indicating the need for expanded education and training. Despite some resistance, there is a growing recognition of the potential benefits of CAD/CAM technology and a trend towards integrating it into regular practice.

## Introduction

The advent of digital technology has brought significant changes to numerous fields, with dentistry being a key participant in this transformative journey [1]. This shift from conventional to digital practices has introduced a new era in dental care characterized by enhanced precision, efficiency, and patient satisfaction.

The integration of digital advancements in dentistry involves the use of various innovative tools and methodologies [2-5]. Among them, computer-aided design and computer-aided manufacturing (CAD/CAM) systems, intraoral scanners, digital radiography, and three-dimensional (3D) printing have emerged as substantial contributors [3]. These technologies have drastically altered the landscape of dentistry, improving diagnostic accuracy, the precision of treatment planning, the execution of complex procedures, and communication between patients and dentists.

With CAD/CAM technology, we can now design and manufacture custom dental restorations such as crowns, veneers, inlays, onlays, and bridges in a single appointment. This not only saves us valuable time but also ensures our patients receive a highly accurate, perfectly fitted restoration [4]. The adoption of 3D printing technology has opened new possibilities for us. We can create precise models of our patients' mouths, custom dental appliances, surgical guides for implant placement, and even prosthetic parts for reconstructive surgery right in our offices. Three-dimensional printing has allowed us to reduce costs, increase efficiency, and continue improving patient care. The use of electronic health records (EHRs) has made it easier for us to store, retrieve, and manage patient information. EHRs have proven invaluable for tracking our patients' medical histories, dental treatments, allergies, and medication use [5]. They also facilitate communication between us and other healthcare providers, improving the overall quality of patient care.

CAD/CAM technology, in particular, has been instrumental in this revolution, enabling the fabrication of custom dental restorations with high accuracy in a significantly reduced time frame [5]. Intraoral scanners and digital radiography have enhanced diagnostic capabilities, enabling more accurate and early detection of dental pathologies. Concurrently, the emergence of 3D printing has reshaped the field of prosthodontics, allowing for the production of precise and personalized dental prosthetics and surgical guides.

Despite the multitude of benefits and conveniences offered by digital dentistry, its adoption and integration into routine practice are not uniform across the board. Various factors, including the high cost of equipment, lack of adequate training and education, resistance to change from traditional methods, and perceived complexity of technology, have been recognized as obstacles. Therefore, this study aims to assess and understand the knowledge and practices of digital dentistry among postgraduate dental students and dental practitioners in the Kingdom of Saudi Arabia.

## Materials and methods

Study setting

The study was conducted in the Kingdom of Saudi Arabia and employed a cross-sectional, closed-ended, mailed questionnaire-based survey to evaluate the knowledge and practices of digital dentistry among postgraduate dental students and dental practitioners. The study was approved by the Institutional Review Board (IRB) of Riyadh Elm University in September 2023, with the approval number FUGRP/2023/330/1020/917 being assigned to this investigation. The inclusion criteria encompassed postgraduate students of different dental colleges and dental practitioners in Saudi Arabia, while undergraduate students, interns, and those who did not consent to participate were excluded.

Sample size calculation

The sample size was estimated using convenience sampling, with a minimum recommended size of 380 participants. This was calculated based on an acceptable margin of error of 5% and a confidence level of 95%, assuming a dentist population of 27,181 and a response distribution of 50% [6]. The sample size was estimated using the Raosoft online sample size calculator (Raosoft, Inc., Seattle, Washington, United States).

Questionnaire utilized

The study instrument comprised a questionnaire with background questions about age, gender, and educational status, along with 21 close-ended questions related to digital dentistry. The first section of the questionnaire captured demographic information, while the second section comprised 21 close-ended questions related to implant dentistry, with four open-ended questions and two close-ended questions assessing participants' demography. Questions one to four determined knowledge and awareness, while questions five to nine assessed the current clinical practice of digital dentistry. Questions 10-21 asked about the participants' awareness of the limitations and benefits of digital dentistry. The questionnaire-based study was cross-sectional and was conducted among postgraduate students and dental practitioners in Saudi Arabia, with ethical approval obtained from the institutional ethical committee.

A self-prepared questionnaire was given to the participants in the format of online forms. The study was explained and informed consent was obtained from the participants before the questionnaire via online forms. The questionnaire was distributed to the subjects, along with instructions, and sufficient time was provided for them to complete the forms. The identity of the participants was kept confidential. This tool consisted of a demographic section followed by 21 close-ended questions that delved into knowledge, clinical practice, and perceptions of the benefits and limitations of digital dentistry, complemented by four open-ended items for detailed responses. The questionnaire was developed in English and validated by a panel of experts in oral medicine, radiology, and digital dentistry to ensure content validity. A pilot test with 30 individuals outside the main sample was conducted to test its reliability, which was confirmed by an acceptable Cronbach's alpha score. Data collection occurred in a controlled setting to minimize external influences and ensure response accuracy. Adjustments made post pilot testing refined the questionnaire's clarity and relevance, ensuring it met the study's needs.

Statistical analysis

To assess reliability, we used the Cronbach's alpha coefficient. We then used Microsoft Excel (Microsoft Corporation, Redmond, Washington, United States) to organize the information from the completed forms. Then, we used IBM SPSS Statistics for Windows, Version 20, (Released 2011; IBM Corp., Armonk, New York, United States) to figure out the percentages and perform descriptive analysis, the Chi-square test, and the analysis of variance (ANOVA) test.

## Results

The descriptive analysis of the demographic characteristics of the study population is detailed in Table 1.

**Table 1:** Demographic characteristics of the assessed sample size

Variable analysed		Frequency (n)	Percentage (%)
Age range (in years)	20-30	215	22.6
	31-40	479	50.3
	41-50	216	22.7
	51-60	43	4.5
Gender	Male	483	50.7
	Female	470	49.3
Region of residence	North	304	31.9
	South	54	5.7
	East	218	22.9
	West	147	15.4
	Central	229	24.0
Educational status	Qualified dentist	559	58.7
	Post-graduate student	394	41.3

Table 2 elucidates the professional characteristics and digital dentistry awareness of the sample population.

**Table 2:** Characteristics pertaining to knowledge and awareness about digital dentistry as assessed in the sample population CAD/CAM: computer-aided design/computer-aided manufacturing

Variable analysed		Frequency (n)	Percentage (%)
Status of dental practice	Private practitioner	326	34.2
	Private practitioner and post-graduate students	142	14.9
	Teaching faculty and private practitioner	216	22.7
	Teaching faculty	60	6.3
	Post-graduate Student	209	21.9
Years of clinical experience	Less than 5 years	231	24.2
	5-10 years	475	49.8
	More than 10 years	247	25.9
Type of dental practice	General dental practitioner	555	58.2
	Specialized practitioner	398	41.8
Were the participants aware of the use of digital technology in dentistry?	Yes	946	99.3
	No	7	.7
Were the participants aware of CAD/CAM technology in dentistry?	Yes	948	99.5
	No	5	.5

**Figure 1:**
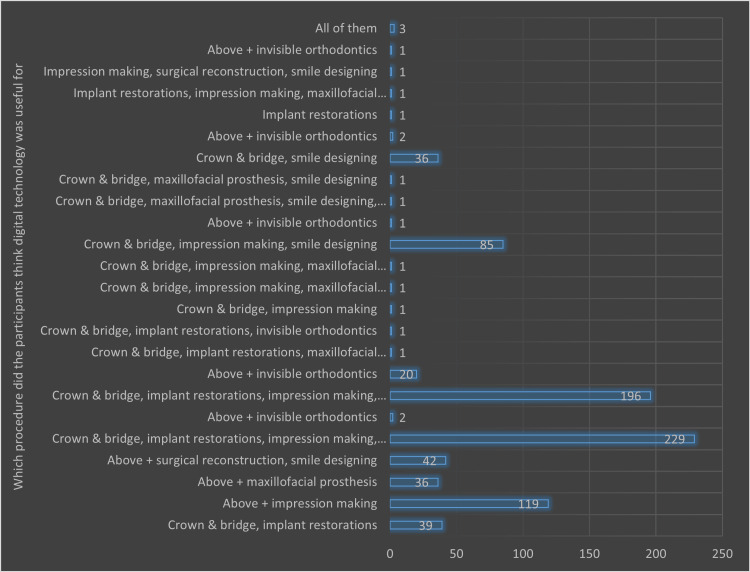
Graphical representation of the opinion of the participants regarding which procedure of digital technology was useful (in terms of the number of responses)

**Figure 2:**
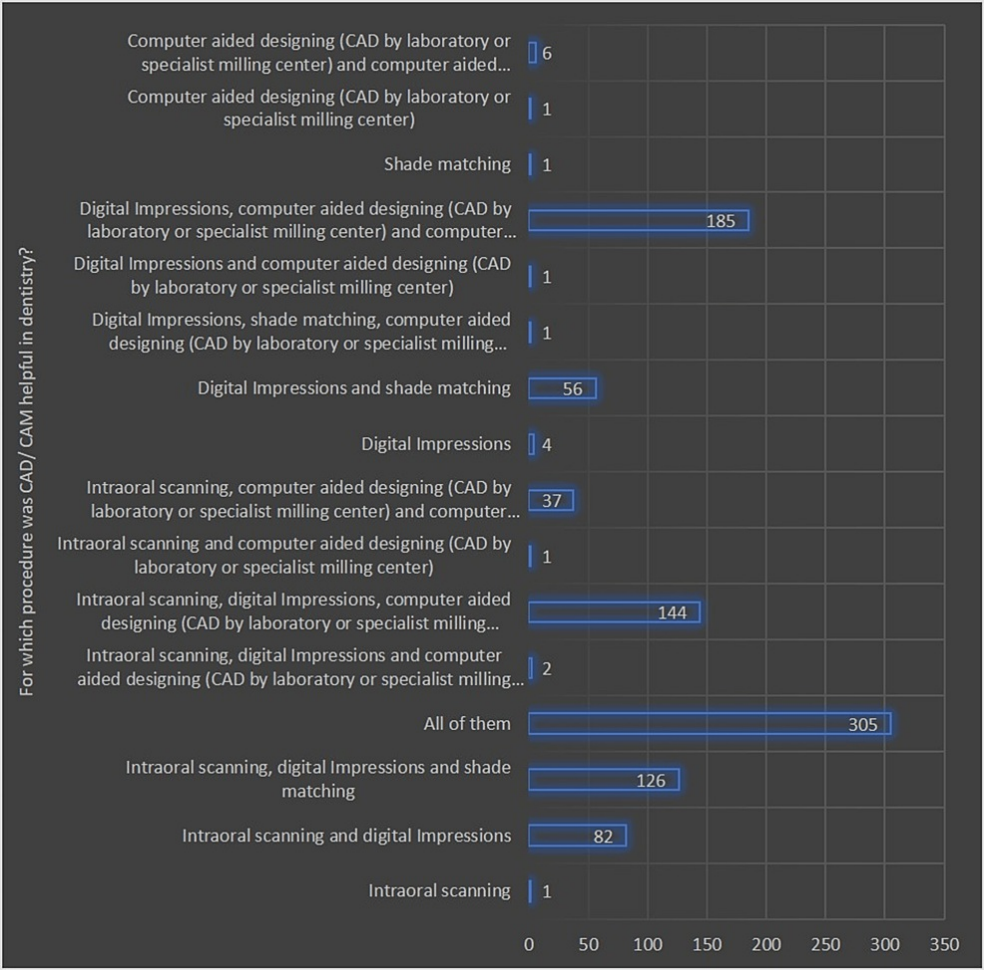
Graphical representation of the opinion of the participants regarding which procedure of CAD/CAM was useful (in terms of the number of responses)

**Figure 3:**
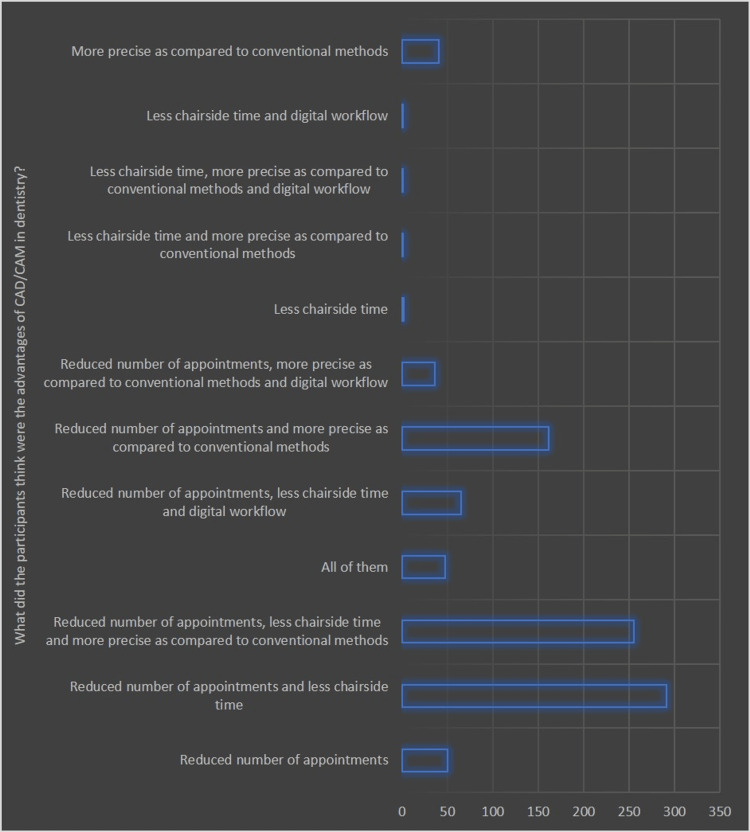
Graphical representation of the opinion of the participants regarding what were the advantages of CAD/CAM (in terms of the number of responses)

Figures 1-3 illustrate the different domains of respondents' understanding and perceptions regarding the clinical applicability of digital technology in dentistry and their awareness of CAD/CAM technology.

In terms of the advantages of CAD/CAM in a clinical scenario, as reported in Table 3, the participants appreciated a wide range of benefits. The most frequently cited advantage, by 20.4% (n=194) of respondents, was a combination of several factors: the elimination of problems associated with impression making, the ability to review and modify the preparation at the same time, immediate data transfer, retrievability of scanned data at any point, and ease in lab authorization and communication.

**Table 3:** Characteristics pertaining to awareness about the current clinical practice of digital dentistry as assessed in the sample population

Variable analysed		Frequency (n)	Percent (%)
What did the participants think were the advantages of CAD/CAM in the clinical scenario?	Solves impression issues	1	.1
	Solves impression issues, allows preparation review and modification	29	3.0
	Above + allows immediate data transfer and data retrievability	1	.1
	Above + eases lab authorization and communication	194	20.4
	Above + ensures accurate and precise fit of restorations/appliances	69	7.2
	All of the above	50	5.2
	Above + ensures accurate orthodontic tooth movement	2	.2
	Solves impression issues, allows preparation review, modification, immediate data transfer, and data retrievability, and ensures accurate fit of restorations/appliances	66	6.9
	Solves impression issues, allows preparation review and modification, and eases lab authorization and communication	41	4.3
	Above + ensures accurate and precise fit of restorations/appliances	132	13.9
	Solves impression issues, allows immediate data transfer and data retrievability, eases lab authorization and communication, and ensures accurate fit of restorations/appliances	1	.1
	Solves impression issues and eases lab authorization and communication	99	10.4
	Above + ensures accurate and precise fit of restorations/appliances	108	11.3
	Solves impression issues and ensures accurate fit of restorations/appliances	44	4.6
	Allows preparation review and modification	1	.1
	Above + allows immediate data transfer and data retrievability	1	.1
	Above + eases lab authorization and communication	55	5.8
	Allows preparation review, modification, and eases lab authorization and communication	54	5.7
	Above + ensures accurate and precise fit of restorations/appliances	1	.1
	Allows immediate data transfer and data retrievability	2	.2
	Eases lab authorization and communication	2	.2

**Figure 4:**
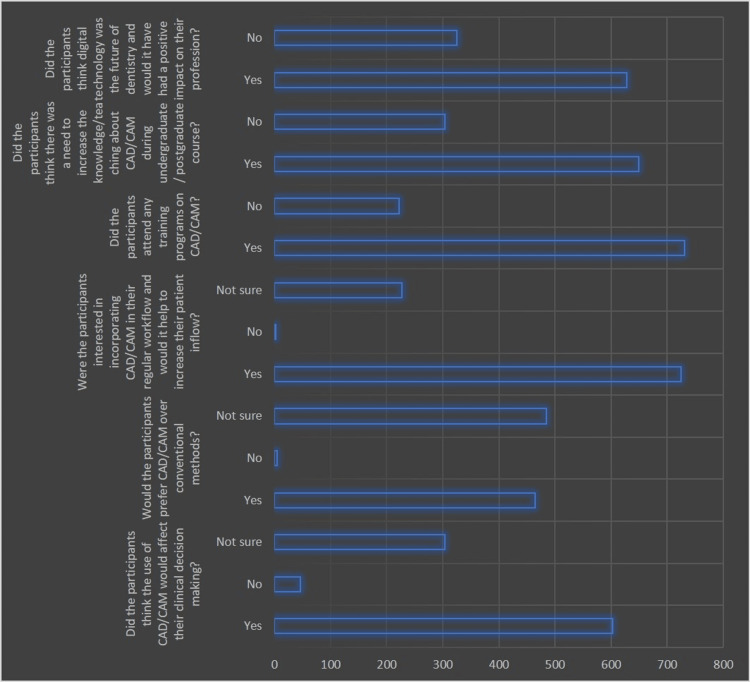
Graphical representation of limitations and benefits of digital dentistry as observed in the included participants CAD/CAM: computer-aided design/computer-aided manufacturing

Figure 4 details participants' opinions on the limitations and benefits of digital dentistry, particularly CAD/CAM systems.

Table 4 presents the results of chi-square tests conducted to analyze the correlations between the educational status of the participants and their knowledge and awareness of digital dentistry.

**Table 4:** Chi-square test results of the correlation between educational status and knowledge and awareness about digital dentistry (where a denotes that the significance (Sig.) value is two-tailed, * indicates a statistically significant result, typically where the Sig. value is less than 0.05 and c suggests that the result is highly significant, often used when the Sig. value is less than 0.01) df: degrees of freedom

Variable analysed		Educational status
Were the participants aware of the use of digital technology in dentistry?	Chi-square	2.129
	df	1
	Sig.	.145^a^
For which procedure did the participants think digital technology was useful?	Chi-square	204.131
	df	24
	Sig.	.000^a,*,c^
Were the participants aware of CAD/CAM technology in dentistry?	Chi-square	.944
	df	1
	Sig.	.331^a^
For which procedure was CAD/CAM helpful in dentistry?	Chi-square	101.148
	df	15
	Sig.	.000^a,*,c^
What did the participants think were the advantages of CAD/CAM in dentistry?	Chi-square	196.592
	df	12
	Sig.	.000^a,*,c^
What did the participants think were the advantages of CAD/CAM in the clinical scenario?	Chi-square	611.709
	df	21
	Sig.	.000^a,*,c^
What were the shortcomings of the use of CAD/CAM in dentistry?	Chi-square	336.598
	df	11
	Sig.	.000^a,*,c^
Which of the following CAD/CAM systems were the participants aware of?	Chi-square	313.983
	df	9
	Sig.	.000^a,*,c^
Which materials did the participants think were regularly used with CAD/CAM?	Chi-square	138.705
	df	7
	Sig.	.000^a,*^
Did the participants think the use of CAD/CAM would affect their clinical decision-making?	Chi-square	126.523
	df	2
	Sig.	.000^*^
Would the participants prefer CAD/CAM over conventional methods?	Chi-square	146.061
	df	2
	Sig.	.000^a,*^
Were the participants interested in incorporating CAD/CAM in their regular workflow and would it help to increase their patient inflow?	Chi-square	84.604
	df	2
	Sig.	.000^a,*,c^
Did the participants attend any training programs on CAD/CAM? Did the participants think there was a need to increase the knowledge/teaching about CAD/CAM during undergraduate/postgraduate courses?	Chi-square	.431
	df	1
	Sig.	.512
Did the participants think digital technology was the future of dentistry and would it have had a positive impact on their profession?	Chi-square	65.436
	df	1
	Sig.	.000^a,*^
Did the participants think the use of CAD/CAM would affect their clinical decision-making?	Chi-square	4.707
	df	1
	Sig.	.030^a,*^

For the question regarding awareness of the use of digital technology in dentistry, the Chi-square value was 2.129, with 1 degree of freedom. The significance level (p-value) was .145, which is greater than the typical threshold of .05, indicating that there was no significant difference in the awareness of digital technology between the two educational groups. In contrast, when asked about the perceived usefulness of digital technology in specific procedures, a significant difference was observed between the groups (Chi-square = 204.131, df = 24, p < .001). This suggests that educational status significantly influences perceptions about the utility of digital technology in various dental procedures.

Similar patterns of significant differences were observed in the understanding of CAD/CAM technology (Chi-square = .944, df = 1, p = .331), the perceived usefulness of CAD/CAM in specific procedures (Chi-square = 101.148, df = 15, p < .001), and the perceived advantages of CAD/CAM in dentistry (Chi-square = 196.592, df = 12, p < .001) and in the clinical scenario (Chi-square = 611.709, df = 21, p < .001). A significant difference was also observed in the perceived shortcomings of CAD/CAM technology (Chi-square = 336.598, df = 11, p < .001). The awareness of different CAD/CAM systems (Chi-square = 313.983, df = 9, p < .001) and the perceived regular materials used with CAD/CAM (Chi-square = 138.705, df = 7, p < .001) also differed significantly between the groups.

Significant differences were observed in the participants' opinions on whether CAD/CAM would affect their clinical decision-making (Chi-square = 126.523, df = 2, p < .001), their preference for CAD/CAM over conventional methods (Chi-square = 146.061, df = 2, p < .001), and their interest in incorporating CAD/CAM into their regular workflow (Chi-square = 84.604, df = 2, p < .001). However, no significant difference was found when asked whether there was a need to increase teaching about CAD/CAM during their education (Chi-square = .431, df = 1, p = .512). Significant differences were also found in the participants' belief in digital technology as the future of dentistry (Chi-square = 65.436, df = 1, p < .001) and in whether the use of CAD/CAM would affect their clinical decision-making (Chi-square = 4.707, df = 1, p = .030).

The first hypothesis that we had devised, asserting similar knowledge between the two groups, was partly rejected due to significant differences in understanding specific aspects of CAD/CAM technology. The second hypothesis, stating no knowledge gap within the entire group, was also rejected due to significant differences in opinions on CAD/CAM's impact on clinical decisions and preferences. However, views on increasing CAD/CAM education were similar. The findings supported the alternate hypotheses, indicating knowledge and practice differences between students and practitioners, as well as knowledge gaps within the group.

## Discussion

Compared to the 40% awareness of CAD/CAM technology in Al-Ibrahim et al. [7], our study revealed a higher awareness (99.5%). Unlike their study, where 71.5% did not use CAD/CAM, a majority (76%) in our study expressed interest in integrating CAD/CAM into their workflow. Both studies agreed on CAD/CAM's clinical usefulness and potential to replace traditional methods.

Nassani et al. [5] showed that 27.2% of participants had a chair-side CAD/CAM system in their practice, a detail not explored in our study. Both studies, however, showed a positive attitude towards CAD/CAM technology and its benefits in time-saving and patient inflow. Our study further revealed a higher percentage (76.7%) of participants had received CAD/CAM training, compared to the 75.4% willingness to learn in Nassani et al.'s study [5].

Our study recorded a high awareness of digital (99.3%) and CAD/CAM technologies (99.5%), which contrasted with that of Alfallaj et al. [8], where 64.4% of dental schools implemented digital technology. Both studies identified barriers to digital dental technology adoption, including cost, lack of knowledge, and training [8]. Radwan et al. [9] reported a 92% agreement on including digital technologies in dental education, corresponding with our 68.1% agreement advocating for enhanced CAD/CAM education. Both studies also noted that a significant portion of participants gained digital technology skills through workshops and courses [9]. However, unlike Radwan et al. [9], our study did not explore generational differences in technology usage.

The integration of digital workflows in dental practices has effectively streamlined and enhanced various clinical and laboratory processes traditionally considered labour-intensive and technique-sensitive [10]. This transformation has addressed several limitations associated with conventional procedures, particularly in terms of quality, labour, and time efficiency [11]. Both dental professionals and patients stand to gain significantly from this paradigm shift. In a study conducted by Saponaro et al. in 2016, it was found that 70% of patients with experience with complete dentures reported a preference for their digitally fabricated replacements over their previous conventionally fabricated dentures [12]. This reveals the perceived improvement in quality brought about by digital dental technologies such as CAD/CAM.

In recent times, there has been an increased recognition of the importance of digital dentistry in Saudi Arabia [13]. A survey investigating the utilization of dental materials for indirect restorations among members of the Saudi Dental Society found that almost a third (29.8%) of respondents were incorporating CAD/CAM systems into their practice [14]. This was substantiated by a later study indicating the presence of chairside CAD/CAM systems in the practices of 27.2% of participating dentists [6]. Contrarily, a similar inquiry among UK dentists revealed a lower adoption rate, with 56% reporting no usage of CAD/CAM components in their practice [15].

The improved quality and safety profile of CAD/CAM digital restorations are being acknowledged more and more, making them a competitive substitute for conventional dental procedures. The superior quality of the resulting restorations and the digital workflow's efficiency are attracting an increasing number of dental practitioners to CAD/CAM technology [16-18]. A United Kingdom (UK) study's results [17] showed that chair-side CAD/CAM restoration quality was not as highly rated. This disparity in perception raises the possibility that more research is necessary. A considerable proportion of dentists using CAD/CAM technology in our study thought that their training in this area was insufficient, which is consistent with the UK survey's findings [15] and points to a possible improvement area for the adoption and application of this technology.

In retrospect, our study had several shortcomings. First, the cross-sectional design of the study made it more difficult for us to determine the causes of the many variables. Second, because our sample may have been skewed towards dentists who had access to and familiarity with digital technologies, it may not have been entirely representative of all dentists in practice. This could have resulted in an overestimation of technology usage and knowledge. Third, because respondents may have inflated or underestimated their skill levels or usage rates, self-reported statistics may have introduced response bias. Fourthly, we were unable to discern between the diverse digital dental technology applications and forms, each of which may have different adoption hurdles and enablers. Lastly, we did not investigate how the adoption of digital technology is influenced by demographic variables like age or years of experience. To give a more comprehensive picture of the adoption of digital technology in dentistry practice, future research should fill in these gaps.

## Conclusions

The assessments of this investigation showed a high degree of knowledge, as almost all participants were aware of the introduction and use of digital technology in dentistry. The overwhelming majority acknowledged the use of CAD/CAM technology, confirming its growing importance in dental practice. However, it was shown that the perceived value of various digital technologies, including CAD/CAM, varies depending on the particular dental treatments that are carried out. The obstacles to the widespread implementation of CAD/CAM technology were also emphasized, although the participants recognized the overall advantages of the technology, such as fewer appointments, shorter chairside times, and more precision over traditional methods. These obstacles included the high price of CAD/CAM technology, a lack of thorough understanding of how to use it, and a preference for conventional techniques. These findings suggest that economic, educational, and preference variables are all important in determining the uptake and integration of such technology. In spite of these obstacles, most participants indicated a willingness to embrace the digital shift and said CAD/CAM technology would have an impact on their clinical judgment. The majority's expressed interest in incorporating CAD/CAM into their routine workflow, a move motivated by the possibility of a rise in patient inflow, further demonstrated this trend. Additionally, the majority of participants had taken part in CAD/CAM training programmes and agreed that undergraduate and graduate courses should offer more instruction in this area, highlighting the role that education plays in easing the shift to digital technology.
